# Contrasting male and female trends in tobacco-attributed mortality in China: evidence from successive nationwide prospective cohort studies

**DOI:** 10.1016/S0140-6736(15)00340-2

**Published:** 2015-10-10

**Authors:** Zhengming Chen, Richard Peto, Maigeng Zhou, Andri Iona, Margaret Smith, Ling Yang, Yu Guo, Yiping Chen, Zheng Bian, Garry Lancaster, Paul Sherliker, Shutao Pang, Hao Wang, Hua Su, Ming Wu, Xianping Wu, Junshi Chen, Rory Collins, Liming Li

**Affiliations:** a Clinical Trial Service Unit & Epidemiological Studies Unit (CTSU), Nuffield Department of Population Health, University of Oxford, Oxford, UK; b Chinese Center for Disease Control and Prevention, Beijing, China; c Chinese Academy of Medical Sciences, Beijing, China; d Qingdao CDC, Qingdao, China; e Zhejiang Provincial CDC, Hangzhou, China; f Heilongjiang Provincial CDC, Harbin, China; g Jiangsu Provincial CDC, Nanjing, China; h Sichuan Provincial CDC, Chengdu, China; i National Center for Food Safety Risk Assessment, Beijing, China; j School of Public Health, Peking University, Beijing 100191, China

## Abstract

**Background:**

Chinese men now smoke more than a third of the world's cigarettes, following a large increase in urban then rural usage. Conversely, Chinese women now smoke far less than in previous generations. We assess the oppositely changing effects of tobacco on male and female mortality.

**Methods:**

Two nationwide prospective studies 15 years apart recruited 220 000 men in about 1991 at ages 40–79 years (first study) and 210 000 men and 300 000 women in about 2006 at ages 35–74 years (second study), with follow-up during 1991–99 (mid-year 1995) and 2006–14 (mid-year 2010), respectively. Cox regression yielded sex-specific adjusted mortality rate ratios (RRs) comparing smokers (including any who had stopped because of illness, but not the other ex-smokers, who are described as having stopped by choice) versus never-smokers.

**Findings:**

Two-thirds of the men smoked; there was little dependence of male smoking prevalence on age, but many smokers had not smoked cigarettes throughout adult life. Comparing men born before and since 1950, in the older generation, the age at which smoking had started was later and, particularly in rural areas, lifelong exclusive cigarette use was less common than in the younger generation. Comparing male mortality RRs in the first study (mid-year 1995) versus those in the second study (mid-year 2010), the proportional excess risk among smokers (RR-1) approximately doubled over this 15-year period (urban: RR 1·32 [95% CI 1·24–1·41] *vs* 1·65 [1·53–1·79]; rural: RR 1·13 [1·09–1·17] *vs* 1·22 [1·16–1·29]), as did the smoking-attributed fraction of deaths at ages 40–79 years (urban: 17% *vs* 26%; rural: 9% *vs* 14%). In the second study, urban male smokers who had started before age 20 years (which is now typical among both urban and rural young men) had twice the never-smoker mortality rate (RR 1·98, 1·79–2·19, approaching Western RRs), with substantial excess mortality from chronic obstructive pulmonary disease (COPD RR 9·09, 5·11–16·15), lung cancer (RR 3·78, 2·78–5·14), and ischaemic stroke or ischaemic heart disease (combined RR 2·03, 1·66–2·47). Ex-smokers who had stopped by choice (only 3% of ever-smokers in 1991, but 9% in 2006) had little smoking-attributed risk more than 10 years after stopping. Among Chinese women, however, there has been a tenfold intergenerational reduction in smoking uptake rates. In the second study, among women born in the 1930s, 1940s, 1950s, and since 1960 the proportions who had smoked were, respectively, 10%, 5%, 2%, and 1% (3097/30 943, 3265/62 246, 2339/97 344, and 1068/111 933). The smoker versus non-smoker RR of 1·51 (1·40–1·63) for all female mortality at ages 40–79 years accounted for 5%, 3%, 1%, and <1%, respectively, of all the female deaths in these four successive birth cohorts. In 2010, smoking caused about 1 million (840 000 male, 130 000 female) deaths in China.

**Interpretation:**

Smoking will cause about 20% of all adult male deaths in China during the 2010s. The tobacco-attributed proportion is increasing in men, but low, and decreasing, in women. Although overall adult mortality rates are falling, as the adult population of China grows and the proportion of male deaths due to smoking increases, the annual number of deaths in China that are caused by tobacco will rise from about 1 million in 2010 to 2 million in 2030 and 3 million in 2050, unless there is widespread cessation.

**Funding:**

Wellcome Trust, MRC, BHF, CR-UK, Kadoorie Charitable Foundation, Chinese MoST and NSFC

## Introduction

For national disease control strategies, and for individual decisions about smoking, what matters is not just current but also future tobacco-attributed mortality rates. China now consumes over a third of the world's cigarettes,^1^ but the increase is too recent for the full effect of current cigarette consumption levels on the hazard per continuing smoker to have emerged.^2^, ^3^ There has been a large intergenerational increase in cigarette smoking by young men, first in urban and then in rural areas. In contrast, there has been a large intergenerational decrease in smoking among women, at least until women born in the 1970s.^4^, ^5^, ^6^, ^7^ So, assessment of future tobacco hazards must allow for urban versus rural and, particularly, male versus female differences in lifelong smoking patterns.

**Research in context**

**Systematic review**

Existing reviews of smoking and death in China include mainly studies established during the 1980s or 1990s.^23^, ^24^ We searched PubMed for further such studies using the terms “smoking” AND “mortality” AND “China”, but found no large studies reported in English. The previous studies mainly involved people born before 1950 who, unlike those born more recently, had not smoked cigarettes persistently since early adulthood, or had smoked forms of tobacco (eg, pipes) that carry a lower risk than manufactured cigarettes. These previous studies could not assess directly the growing risks of tobacco in the present century. For, the hazards among smokers depend importantly not only on recent smoking patterns, but also on patterns in early adult life,^2^, ^13^, ^14^ and consumption of substantial numbers of cigarettes from early adulthood used to be uncommon, particularly in rural areas. Future hazards can be assessed only by studies of large numbers who have smoked cigarettes ever since early adulthood. Our two nationally representative studies can help to assess how the epidemic of smoking in mainland China has developed, and how it will evolve.

**Interpretation**

There are contrasting male and female trends. Among women, tobacco-attributed mortality is currently about 5%, 3%, 1%, and <1% of all mortality in those born in the 1930s, 1940s, 1950s, and since 1960. Female tobacco-attributed mortality is likely to fall as the pre-1950 generation, in which some women smoke, is replaced by the next generation, in which far fewer do so. Among men, the situation is opposite; the proportion of male deaths from smoking has been increasing, first in urban and then in rural areas, and already by 2010 tobacco accounted for a quarter of urban male deaths at ages 40–79 years. Among urban male smokers who started to smoke cigarettes (rather than other forms of tobacco) before age 20 years, which is the uptake pattern that is now typical among young men throughout China, overall mortality is already twice as great as among otherwise similar men who never smoked. As the epidemic matures, first in urban and then in rural areas, and the population grows, Chinese tobacco deaths will rise from about 1 million in 2010, to 2 million in 2030, and 3 million in 2050, unless there is widespread cessation.

Assessment of current and future tobacco hazards must also allow for other changes in mortality. Communicable disease mortality is decreasing steeply and non-communicable disease mortality is also decreasing, albeit more slowly.^8^, ^9^ This health transition is continuing, so all-cause mortality is falling in children and in adults, despite the net effects of changes in smoking and in adiposity. At 1970 Chinese rates, half would die before age 70 years (including 10% before age 5 plus another 10% before age 50). At 2010 rates, however, only a quarter would die before age 70 years (including only 1% before age 5 plus another 4% before age 50).^9^ These improvements are likely to continue (except perhaps in male smokers), due to better treatment and reductions in many other causes of disease.

The Chinese population has stabilised below age 50, but at ages 60–79 years it will double from 2010 to 2030, then stabilise.^10^ The rising over-50 population and falling age-specific death rates affect projections of tobacco-attributed mortality, and of the eventual risks for those smokers who, as has become usual for men in urban and then rural China, started substantial cigarette usage before age 20 years. The hazards currently seen in urban men who started cigarette smoking before age 20 years are particularly important, as they foreshadow what nationwide smoker versus non-smoker mortality rate ratios (RRs) will eventually become.^2^, ^3^

Successive nationally representative epidemiological studies, conducted some time apart, can help to monitor and predict trends in tobacco-attributed mortality.^11^, ^12^, ^13^, ^14^, ^15^ We report two large studies in China, one monitoring deaths from 1991–99 (mid-year 1995),^16^ and the second monitoring deaths 15 years later, from 2006–14 (mid-year 2010).^17^, ^18^ Changing smoker versus non-smoker RRs between the two studies show how the epidemic is evolving. The baseline survey of the second study (which was more detailed than that of the first) yields data on intergenerational changes in smoking patterns among urban and rural men and women, and prospective follow-up of mortality yields the RRs associated with each pattern, including the particularly informative hazards among urban men who have smoked cigarettes since before age 20 years.

## Methods

### Study populations

After a pilot phase in 1990–91, the first nationwide prospective study (Chinese Prospective Smoking Study [CPSS]) had its main recruitment phase from April 9, to Dec 31, 1991, with follow-up to Dec 31, 1999 (mid-year 1995). The second (China Kadoorie Biobank [CKB] study) recruited from June 25, 2004, to July 15, 2008, with follow-up to Jan 1, 2014 (mid-year 2010). The designs and methods of both studies have been described previously.^16^, ^17^, ^18^

In the first, 225  721 men were recruited from 45 study areas, chosen at random from 145 nationally representative Disease Surveillance Points (DSPs). A typical DSP monitors cause-specific deaths in 50 000–100 000 residents in five to ten nearby residential units (groups of rural villages or urban street committees). In each study area (22 rural, 23 urban), two or three such units were randomly selected and all men aged 40 years or older were identified through local residential records and invited to take part; about four-fifths participated (including 219 893 at ages 40–79 years). In local study clinics, trained health workers measured blood pressure, height, weight, and peak expiratory flow rate and administered a standardised questionnaire on demographics, education, occupation, smoking, drinking, diet, and self-reported medical history. Survey records were handwritten on paper, with subsequent data entry double-punched.

In the second study, 210 222 men and 302 669 women were recruited in 2004–08 from ten diverse locations across China, four urban and six rural (or semi-rural in the case of Suzhou). These study areas chosen from the DSPs to span a range of socioeconomic levels, risk factor patterns, and disease patterns. The set of all DSPs is nationally representative, and after excluding a few with organisational difficulties, the choice of ten study sites from the remaining DSPs was made centrally, knowing local characteristics and cause-specific death rates. The exact choice of areas was decided carefully, aiming successfully to retain geographic and social balance and balance across deciles of mortality from major diseases, so the set of ten study sites should still be approximately nationally representative. All 1 801 167 registered residents of age 35–74 years were identified through local records and invited to survey clinics, and 500 223 (28%) participated; another 12 668 just outside this age range also participated. As a substantial minority of registered residents would actually have been living elsewhere, we estimate that about a third of the invitees actually living in the study areas participated. Trained health workers took blood for long-term storage; measured height, weight, waist, hips, bioimpedance, blood pressure, heart rate, and lung function; and administered laptop-based questionnaires on tobacco, alcohol, diet, indoor air pollution, physical activity, education, socio-demographic status, medical history, and female reproductive history.^17^

### Assessment of smoking

In both studies, questions about *s*moking included frequency, type, amount (current and past), age first began, age stopped, and main reason for cessation (already ill or stopped by choice), with additional information in the second study on inhalation and exhaled CO (MicroCO meter, Carefusion, San Diego, CA, USA).^19^ Regular smokers used one or more cigarettes (or ≥1 g tobacco) daily for at least 6 months. Of smokers who had stopped (≥6 months), about half had done so because they were ill, and (to avoid bias) were still counted with smokers in the main analyses; the remainder, who are described as having stopped by choice, helped assess the effects of cessation.

### Mortality follow-up

Cause-specific mortality in both studies was monitored through DSP death registries,^20^ and checked annually against local residential records, with active confirmation of survival through street committees or village administrators.^18^ In the second study, deaths were also monitored through the new nationwide health insurance system (yielding few additional cases). Causes were checked against any available medical records. For the few deaths (about 5% in both studies) without recent medical attention, standardised procedures determined probable causes from symptoms or signs described by informants (usually family). Deaths were coded by trained staff, blind to baseline data, using International Classification of Diseases (ICD)-9 in the first study and ICD-10 in the second (appendix p 3). At ages 40–79 years, each study had about 25 000 deaths.

### Statistical analysis

Cox regression yielded multi-covariate-adjusted smoker versus never-smoker RRs at ages 40–79 years. Pack-years were not used, as ten cigarettes per day for 40 years may have effects very different from those of 20 cigarettes per day for 20 years. Analyses were stratified for location (first study 45 areas, second study ten areas) and 5-year age-at-risk groups, and adjusted for education (tertiary, secondary, primary, or none completed) and alcohol consumption (never, occasional, or ever-regular).

The RR for each smoking category is accompanied by a CI derived only from the variance of the log risk in that one category. Hence, each RR, including that for the reference group, is associated with a group-specific CI that can be thought of as reflecting the amount of data only in that one category.^21^ The 95% group-specific CI for RR is (RR/T, RR×T), where T=exp (1·96√v) and v is the variance of the log risk.

If the reference group with RR=1 and another group with RR=R have, respectively, group-specific CIs (a, b) and (x, y), then the CI for R that allows for the variation in both of the groups is (√ [xy/k], √ [xyk]), where log (k) is given by √(log^2^ [y/x] + log^2^ [b/a]); since k>y/x, this CI is wider than (x, y).

If RR is causal, the fraction of all deaths in the population that is attributed to smoking (ie, the population-attributed fraction; PAF) is P(RR-1) divided by RR, P being the prevalence of smoking among those dying of the relevant cause.^22^ Setting P=1 yields (RR–1) divided by RR, the fraction of the mortality among smokers that is attributed to smoking. Analyses used SAS version 9.3.

### Role of the funding sources

The funders of the study had no role in study design, data collection, data analysis, data interpretation, or writing of the report. ZC, RP, and LL had full access to all the data in the study and had final responsibility for the decision to submit for publication.

## Results

**Figure 1:**
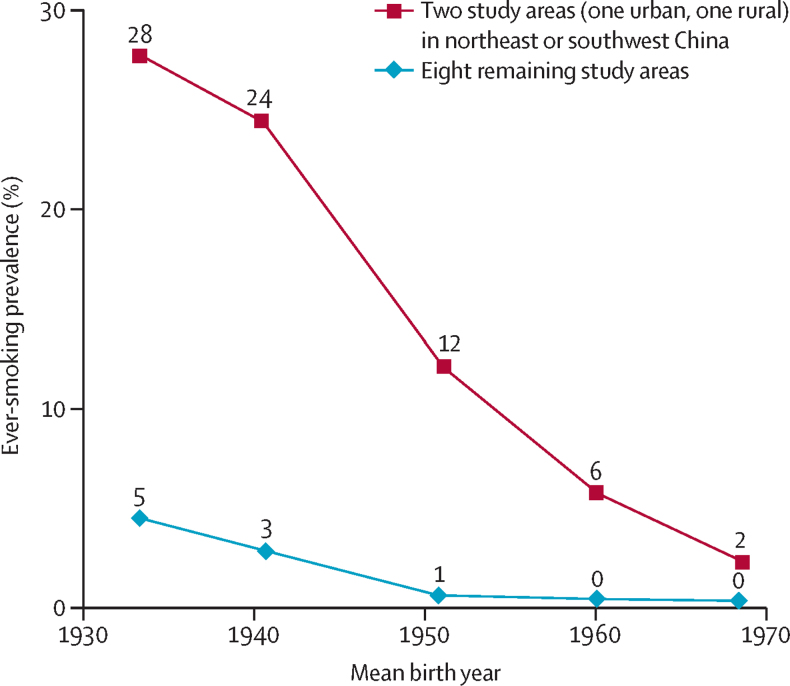
Chinese female smoking uptake rate by year of birth and locality 300 000 women seen in ten study areas in about 2006, with birth years grouped as: before 1935, 1935–44, 1945–54, 1955–64, and 1965 or later. The two areas where many older women smoked are in Harbin (urban northeast China) and Sichuan (rural southwest China). Taking all ten areas together, the prevalences of ever-smoking among women born in the 1930s, 1940s, 1950s, 1960s, and 1970s were, respectively, 10%, 5%, 2%, 1%, and <1% (3097/30 943, 3265/62 246, 2339/97 344, 926/94 772, and 142/17 161).

Women were included only in the second study. Of 302 669 women enrolled in 2004–08, only 3·2% were ever regular smokers, including 0·5% who stopped by choice. Smoking uptake decreased steeply across successive generations of women. Hence, there were much lower prevalences among those born in more recent decades (figure 1). Taking all study areas together, the prevalences of smoking among women born in the 1930s, 1940s, 1950s, and since the 1960s were 10%, 5%, 2%, and 1% (see figure 1 legend). In most locations, few women smoked, although in urban Harbin (northeast China) and rural Sichuan (southwest China), appreciable numbers of older women did so. Both in these two locations and elsewhere, the prevalence was about tenfold lower in women born in the 1960s (age about 40 years in 2006) than in women born in the 1930s (age about 70 years in 2006). On average, female smokers had started at age 27 years and currently smoked about 10 g/day (ten cigarette equivalents/day, though not necessarily as cigarettes).

During 2·0 million woman-years of follow-up (mid-year 2010), 9934 women died at ages 40–79 years. Female mortality rates were significantly related to smoking for all causes (RR 1·51, 1·40–1·63) and for lung cancer, ischaemic heart disease, and chronic obstructive pulmonary disease (COPD) (appendix p 4). The proportional increase in overall mortality among smokers was greater in urban (RR 1·72, 1·52–1·96) than in rural (RR 1·40, 1·27–1·54) women, but numbers were too small for subdivision by smoking patterns. If these associations are causal, then about a third (0·51/1·51) of all deaths of female smokers were due to tobacco. As, however, few women smoked, the fraction of female mortality attributed to smoking was only 3% for deaths at ages 40–79 years (and 1·1%, 2·7%, and 5·2% for deaths at ages 40–59, 60–69, and 70–79 years). This dependence on age can also be expressed in terms of decade of birth: smoking caused about 5%, 3%, 1%, and less than 1% of all deaths among Chinese women born in the 1930s, 1940s, 1950s, and since 1960. The prevalences of smoking in these four birth cohorts were 10%, 5%, 2%, and 1%, respectively.

**Figure 2:**
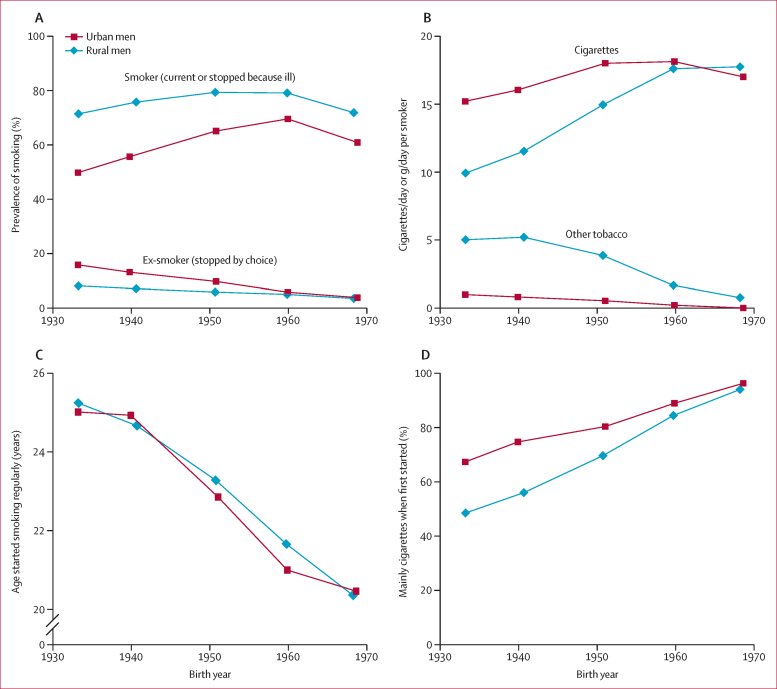
Urban and rural Chinese male smoking patterns, by year of birth—prevalence, consumption, age started, and tobacco type smoked initially 210 000 men seen in about 2006 (at the 2004–08 baseline survey for the second prospective study). Prevalence of smoking (A); amount smoked per day when last smoked (B); Mean age started smoking regularly (C); and percentage of all smokers who used cigarettes when first started (D). To avoid reverse causality biasing the apparent effects of smoking and of cessation, in panel (A) and in the main analyses, the few men who had stopped smoking because they were ill are combined with the continuing smokers, leaving the ex-smokers who had stopped by choice. The overall proportions of men who had stopped because they were ill were 2·16%, 2·47%, 2·19%, 1·08% and 0·12% for those born during the 1930s, 1940s, 1950s, 1960s, and 1970s respectively.

Among men, the smoking patterns were very different. The first of these two prospective studies began about 15 years before the second (1991 *vs* 2006), so those in it had, on average, an earlier year of birth (1938 *vs* 1954) and were less likely to have smoked only cigarettes when they started smoking, or last smoked (appendix p 5). The prevalences of ever-smoking among men in the two studies were similar, and did not depend strongly on age. In the second study, 68% were smokers (including those who had stopped because they were ill) and another 7% were ex-smokers (described as stopping by choice). Figure 2, from the baseline survey for the second study, describes smoking patterns by birth year. Male smokers born in 1970 had started around age 20 years and used only cigarettes, but male smokers born in the 1930s (an earlier generation) had started around age 25 years, smoked other tobacco types, and (not shown) were slightly less likely to inhale deeply. The proportion of smokers who had used cigarettes throughout adulthood was higher in urban than in rural men.

Male deaths at ages 40–79 years numbered 25 548 in the first study and 14 241 in the second. In both, smokers' mortality rates were significantly elevated (first study: RR 1·17, 1·14–1·21; second study: RR 1·33, 1·28–1·39; table 1). So, although the studies were only 15 years apart (mid-years of follow-up 1995 and 2010), the proportional excess mortality among smokers (RR-1) had approximately doubled. Within each study, the RRs for all-cause mortality were not as extreme in rural as in urban men (first study: rural RR 1·13, 1·09–1·17 and urban RR 1·32, 1·24–1·41; second study: rural RR 1·22, 1·16–1·29 and urban RR 1·65, 1·53–1·79). Apart from the rural versus urban difference, the RRs in the second study differed little across study sites (appendix p 6). If the associations of smoking with death are largely causal, then the proportion of all male deaths at ages 40–79 years attributed to smoking rose between 1995 and 2000 from about 11% in the first study (PAF 9% rural, 17% urban) to about 18% in the second study (PAF 14% rural, 26% urban). If the PAF rose from 11% in 1995 to 18% in 2010, it will be at least 20% by the mid-2010s. Hence, smoking will cause about 20% of all male deaths at ages 40–79 years during the 2010s (ie, 2010–19).

Similarly, the proportional excess mortality among smokers (ie, RR-1) increased substantially between 1995 and 2010 for lung cancer (first study: RR 1·95, 1·68–2·26; second study: RR 2·58, 2·17–3·05), all vascular disease (first study: RR 1·12, 1·07–1·18; second study: RR 1·24, 1·16–1·33), and COPD (first study: RR 1·28, 1·20–1·36; second study: RR 1·62, 1·38–1·90).

**Figure 3:**
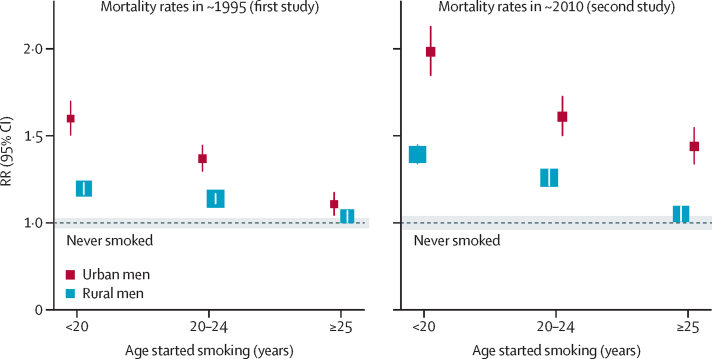
All-cause smoker versus never-smoker mortality rate ratio (RR) among urban and rural Chinese men in two prospective studies, by time period (about 1995 or about 2010) and by age started smoking regularly Each study followed about 200 000 men. Each group-specific CI (including that for never-smokers, given by the width of the shaded strip) reflects the variance of the log risk in that one group, so comparisons of RRs use variances from more than one group.

Figure 3 shows, for both studies, the urban and rural RRs for overall mortality by age started smoking regularly. In each case, men who had started before age 20 years (mean age 17 years) were at substantially greater risk than were those who had started at ages 20–24 years (mean age 21 years). Differences in the reported ages at which men had started smoking regularly did not, however, suffice to explain the more extreme RRs in the second study, as even within each category of age started, the RR was substantially more extreme in the second study (table 1).

Among urban male smokers in the second study who had started before age 20 years (as is now typical throughout urban and rural China), most had always used manufactured cigarettes, and their all-cause mortality rate was double that of non-smokers (RR 1·98, 1·79–2·19). This suggests that about half (0·98/1·98) of their deaths were due to tobacco. This proportion could well rise still further. The few urban men who had started before age 15 years were at even greater risk (RR 2·64, 2·19–3·19).

The remaining analyses are given separately for urban and rural men in the two studies. The discussion particularly emphasises findings in the second study for urban smokers, among whom the RR was 1·65 (1·53–1·79) for all causes, 2·98 (2·28–3·89) for lung cancer, 1·63 (1·39–1·90) for ischaemic stroke or ischaemic heart disease, and 4·61 (2·75–7·73) for COPD; the RRs were even more extreme for men who had started before age 20 years (table 1). Smokers also had elevated mortality from some other diseases (appendix p 4), including stomach and oesophagus cancer, which are still major causes of death in some parts of China. In rural men, the RRs were generally less extreme, especially for vascular disease and COPD.

For the same disease groupings, table 2 shows urban and rural RRs in both studies by amount last smoked (<15, 15–24 and ≥25 g/day, adding together cigarettes per day and g per day of tobacco). It is only the RRs (not the absolute mortality rates) that are compared between areas and studies, as in table 1. For smokers in the second study, among urban men there were highly significant dose-response relationships for mortality from all causes, lung cancer, ischaemic stroke and ischaemic heart disease (each trend p<0·0001), and COPD (trend p=0·0021), but in rural men, the dose-response relationships among smokers (ignoring the non-smokers) were significant only for lung cancer, a disease with little time between symptom onset and death. The lack of a dose-response relationship for COPD (a major cause of death in rural areas) may reflect biases from reverse causality, whereby COPD symptoms could reduce the amount smoked. For all four disease groupings, tests for trend that included the never-smokers as having zero dose were highly significant in urban and rural areas in both studies (16 trend tests each p<0·0001; table 2).

**Figure 4:**
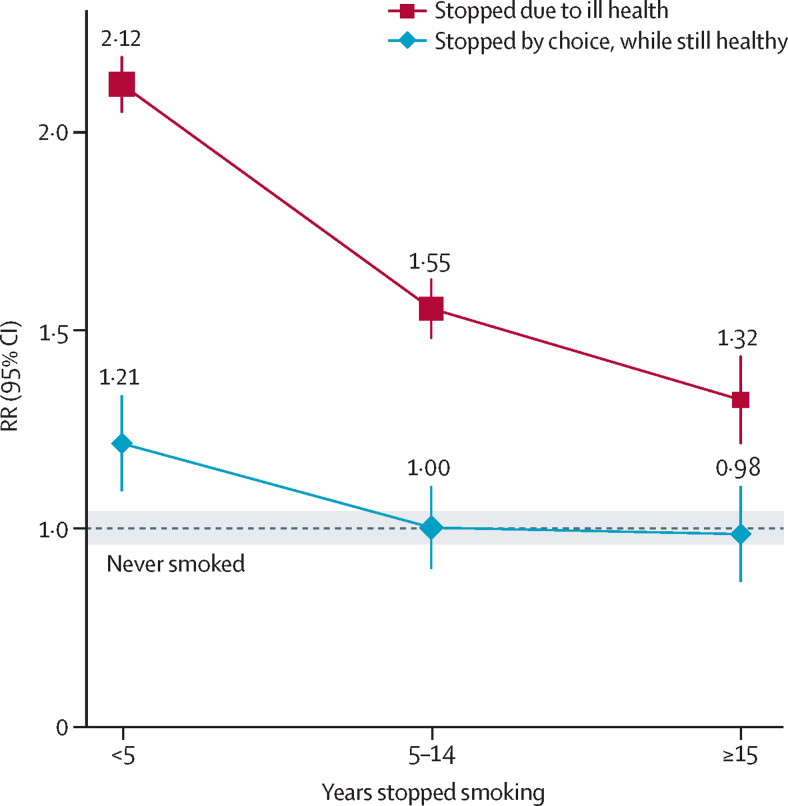
Ex-smoker versus never-smoker all-cause mortality rate ratio (RR), by years stopped smoking and reason stopped, for men in the second study Each group-specific CI (including that for never-smokers, given by the width of the shaded strip) reflects the variance of the log risk in that 1 group, so comparisons use variances from more than one group.

The proportion of male smokers who had stopped by choice rose appreciably over the 15 years from 1991 to 2006, from only 3% (4306/160 971) in the first baseline survey to 9% (14 080/156 313) in the second, and stopping by choice avoided nearly all the excess risk that would have been seen at the mortality rates of continuing smokers (figure 4). Among ex-smokers who had stopped by choice, the RR for all-cause mortality was 1·02 (0·95–1·10), and it attenuated with quitting duration. For men who had stopped by choice less than 5 years, 5–14 years, and 15 or more years before baseline, the RRs were 1·21 (1·07–1·37), 1·00 (0·90–1·11), and 0·98 (0·87–1·11) (trend p=0·01). These findings were not materially changed by inclusion of the first-study results (appendix p 7). Numbers who stopped by choice and died from specific conditions were too small for separate analysis. Among men who had stopped because they were already ill, the protective effects of quitting cannot be assessed straightforwardly. For, even if cessation is substantially protective, the illness that had made them stop could still cause them to be at misleadingly elevated risk (figure 4).

The smoker versus non-smoker RRs for lung cancer in these studies are much less extreme than in recent US prospective studies (appendix p 8–10), but the absolute excess lung cancer mortality in Chinese smokers is still substantial, as Chinese people have much higher non-smoker lung cancer rates than Americans do, especially in later middle age.^3^ Within China, never-smoker lung cancer rates fell slightly between the two prospective studies, whereas the smokers' lung cancer rates rose, resulting in greater RRs in the second study.

## Discussion

Among Chinese men, tobacco-attributed mortality has grown considerably since the 1990s, and during the 2010s, smoking will cause about 20% of all male deaths at ages 40–79 years, up from only about 10% in the early 1990s. Moreover, the mortality rate ratio of 2 already seen among urban male smokers who started before age 20 years (the uptake pattern now typical in both urban and rural China) suggests that about half of these men's deaths were caused by smoking. This mortality rate ratio of 2 is still increasing, foreshadowing substantially greater future hazards for Chinese men (panel). Also, although this was not assessed in the present report, tobacco causes many non-fatal disease episodes, and much disability.

Among Chinese women, however, tobacco-attributed mortality is much smaller, and is currently still falling. Fewer women than men are smokers, and there was a large intergenerational decrease in the female prevalence of smoking, from about 10% among women born in the 1930s down to only about 1% among women born around 1970, which has been confirmed in an independent nationwide survey.^6^ Hence, although the present study suggests that the hazard per smoker is comparable for men and women, as did previous studies,^13^, ^14^, ^23^ tobacco-attributed mortality in the entire female population is low, and will fall further by 2030 as the present generation of women in which few smoke is succeeded by a generation in which even fewer smoke. (A recent WHO report mistakenly suggested, using methods^3^ inappropriate for China, that male and female tobacco-attributed proportions of deaths are similar;^24^ this is impossible, given the 20-fold difference in smoking prevalence.) But, this favourable trend may cease (with women born around 1980 and now in their thirties being the least exposed generation), as tobacco use by adolescent females has recently begun to increase in some parts of China.^7^ This underlines the danger that, as in many Western countries, social changes may well lead young Chinese women to start smoking. It will, however, take decades, probably until after 2050, for an increase in female smoking to cause a substantial increase in mortality, and over the next 20 years, while tobacco-attributed deaths increase among Chinese men, they should decrease among Chinese women, perhaps from about 3% of all female deaths now to less than 1% by the 2030s.^2^

A few large studies have provided reasonably robust evidence about the hazards of smoking in specific Chinese male populations, but most of the people who died in them were born before 1950,^16^, ^25^, ^26^, ^27^, ^28^, ^29^ so the smokers were at limited risk, as in the USA 60 years ago.^11^, ^14^ Some studies^29^, ^30^ have attempted to assess the extent of the Chinese epidemic in the early 2000s, but they involved cohorts established decades ago of men born before 1950, or used data from atypical regions (eg, Shanghai) where manufactured cigarettes have long been available.^31^

In China, cigarette consumption became widespread earlier in urban than in rural areas, mainly because of limited rural availability (and, until recent decades, affordability) of cigarettes.^32^ Hence, the hazard associated with a given current smoking pattern is more extreme in urban than in rural areas. However, this urban versus rural difference is likely to diminish, or even be reversed, over the next few decades, because rural men born after the 1960s not only tended to start at the same age as urban men and to smoke only cigarettes, but also had a somewhat higher smoking prevalence (figure 2). As earlier generations of urban and rural men get replaced by generations who have smoked cigarettes persistently since early adulthood, tobacco-attributable risks in middle age may soon reach those seen in many Western populations, as has almost happened in the subgroup of urban men who started smoking before age 20 years in the present study (and in Hong Kong, where cigarette use peaked about 20 years earlier than in mainland China^33^).

For the chief diseases by which tobacco causes death, there are large quantitative differences between China and elsewhere, between urban and rural China, and between past and future decades.^2^ In many Western populations, tobacco used to cause far more deaths from vascular than from respiratory disease,^11^, ^12^, ^13^, ^14^, ^15^ whereas in China the opposite is true, especially in rural areas. Although the RRs for COPD, lung cancer, and stroke may at present be smaller than in many Western populations, the absolute risks associated with smoking are not, as Chinese non-smoker death rates are high. Indeed, 1980s lung cancer mortality among Chinese never-smokers was more than three times that in US never-smokers, perhaps due partly to indoor air pollution from heating and cooking.^2^ While US never-smoker lung cancer rates have remained roughly constant over the past half-century,^3^ those in Chinese never-smokers seem to be decreasing while those in smokers are increasing, causing increasing smoker versus non-smoker lung cancer mortality rate ratios.

Absolute mortality rates are likely to be lower in prospective studies than in the general population. Hence, to estimate absolute numbers of tobacco-attributed deaths in China in 2010, the smoking-attributed fractions of all deaths in our second prospective study have been applied to independent estimates of male and female cause-specific numbers of deaths in mainland China^34^ at ages 35–69, 70–79, and 80 years or older (appendix p 4). This shows that there were about 1 million smoking-attributed deaths in 2010 (840 000 male, 130 000 female; table 3), mainly from diseases already known to be affected by smoking (lung cancer, ischaemic heart disease, ischaemic stroke, COPD, and other neoplastic, vascular, and respiratory conditions).^3^, ^11^ Counterbalancing the increasing RRs for all-cause mortality, age-specific under-70 mortality rates in China are decreasing due to many social, occupational and health-care changes, falling by about 15% during 2000–10,^9^ so the absolute death rate from smoking is not increasing as fast as would be suggested just by the increasing RRs.

About two-thirds of young Chinese men become cigarette smokers in early adult life. Unless they stop, the present study suggests that at least half of them will eventually be killed by their habit, and future studies may well show that a somewhat greater proportion will be killed by it. The first generation of men to experience the full hazards will probably be those born during the 1970s or 1980s, who reached adulthood when nationwide cigarette consumption was high. Conversely, this may well be the least exposed female generation.

China's 2030 sustainable development goals include reducing non-communicable disease mortality by a third, and monitoring the changes. If current smoking patterns persist, then as the smoker versus non-smoker RRs increases, mortality from other causes decreases, and the over-60 population doubles, Chinese tobacco deaths are likely to rise from 1 million in 2010 to about 2 million in 2030. Nowadays, in China more than 6 million young men a year begin smoking. If most persist, and (as in the USA^14^ and UK^12^, ^13^) smokers eventually have more than double the non-smoker mortality rates, there will in 2050 be about 3 million Chinese tobacco deaths, when those born in 1970 reach age 80 years. Although continuation of our second prospective study will monitor how the epidemic develops over the next decade or two, large new prospective studies of people born after 1970 will be needed to continue monitoring it thereafter. Fortunately, China's nationally representative household surveys regularly record smoking and the reasons for smoking cessation, and electronic linkage of this information with routine mortality records should allow reliable monitoring of the evolution of the epidemic for many decades to come.

Avoiding uptake of smoking by young people will greatly reduce tobacco deaths in the second half of the century. Moreover, stopping before the onset of life-threatening illness is remarkably protective, and an increasing proportion of smokers have stopped by choice (9% in 2006 *vs* only 3% in 1991). With effective measures to accelerate cessation, the growing epidemic of premature death from tobacco can be halted and then reversed, as in other countries. Widespread smoking cessation offers China one of the most effective, and cost-effective,^35^ strategies to avoid disability and premature death over the next few decades.

## Figures and Tables

**Table 1:** Age started smoking regularly versus cause-specific mortality rate ratio (RR) among urban and rural men in about 2010 (second study) and about 1995 (first study)

		**Breath CO, ppm**	**All causes**		**Lung cancer**		**Ischaemic stroke or ischaemic heart disease**		**COPD**	
			Number of deaths	RR* (95% group-specific CI)	Number of deaths	RR* (95% group-specific CI)	Number of deaths	RR* (95% group-specific CI)	Number of deaths	RR* (95% group-specific CI)
**Urban men, about 2010 (2006–14)**										
Age started smoking (mean), years										
	Age <20 (16·9)	16·5	843	1·98 (1·85–2·13)	127	3·78 (3·15–4·54)	200	2·03 (1·75–2·35)	43	9·09 (6·60–12·50)
	Age 20–24 (21·0)	15·2	797	1·61 (1·50–1·73)	127	3·17 (2·67–3·77)	182	1·50 (1·30–1·74)	24	3·89 (2·62–5·79)
	Age ≥25 (30·4)	12·6	754	1·44 (1·34–1·55)	96	2·23 (1·82–2·73)	196	1·49 (1·29–1·72)	24	2·89 (1·93–4·32)
All smokers		14·9	2394	1·65 (1·59–1·73)	350	2·98 (2·66–3·33)	578	1·63 (1·49–1·77)	91	4·61 (3·71–5·71)
Non-smokers		4·5	924	1·00 (0·94–1·07)	69	1·00 (0·79–1·27)	242	1·00 (0·88–1·14)	18	1·00 (0·62–1·60)
Trend p value†				<0·0001		0·0002		0·0032		<0·0001
**Rural men, about 2010 (2006–14)**										
Age started smoking (mean), years										
	<20 (16·8)	15·1	2710	1·39 (1·34–1·45)	271	2·91 (2·58–3·29)	334	1·37 (1·23–1·53)	315	1·88 (1·68–2·10)
	20–24 (21·2)	13·6	2788	1·26 (1·21–1·31)	275	2·45 (2·17–2·75)	397	1·35 (1·23–1·49)	261	1·41 (1·24–1·59)
	≥25 (31·3)	11·8	2434	1·05 (1·01–1·10)	177	1·63 (1·41–1·90)	347	1·04 (0·93–1·16)	233	1·07 (0·94–1·22)
All smokers		13·6	7932	1·22 (1·20–1·25)	723	2·30 (2·13–2·48)	1078	1·24 (1·17–1·32)	809	1·41 (1·31–1·51)
Non-smokers		6·0	2031	1·00 (0·96–1·05)	92	1·00 (0·81–1·23)	316	1·00 (0·89–1·12)	172	1·00 (0·86–1·16)
Trend p value†				<0·0001		<0·0001		0·0005		<0·0001
**Urban men, about 1995 (1991–99)**										
Age started smoking (mean), years										
	<20 (16·7)	..	1158	1·60 (1·51–1·70)	142	3·20 (2·69–3·80)	178	1·58 (1·36–1·84)	194	1·82 (1·57–2·21)
	20–24 (21·2)	..	1358	1·37 (1·30–1·44)	130	2·31 (1·95–2·75)	208	1·37 (1·19–1·57)	209	1·43 (1·25–1·64)
	≥25 (30·6)	..	1136	1·11 (1·04–1·17)	97	1·73 (1·42–2·12)	178	1·17 (1·01–1·36)	186	1·16 (1·00–1·34)
All smokers		..	3652	1·32 (1·28–1·37)	369	2·32 (2·08–2·59)	564	1·35 (1·23–1·47)	589	1·42 (1·30–1·55)
Non-smokers		..	1381	1·00 (0·95–1·06)	82	1·00 (0·80–1·25)	240	1·00 (0·87–1·14)	197	1·00 (0·87–1·15)
Trend p value†				<0·0001		<0·0001		0·0052		<0·0001
**Rural men, about 1995 (1991–99)**										
Age started smoking (mean), years										
	<20 (16·8)	..	5107	1·20 (1·16–1·23)	236	1·98 (1·73–2·26)	435	1·12 (1·02–1·23)	1373	1·40 (1·33–1·48)
	20–24 (20·9)	..	6215	1·14 (1·11–1·17)	280	1·82 (1·62–2·05)	605	1·17 (1·08–1·27)	1573	1·28 (1·22–1·34)
	≥25 (29·6)	..	3901	1·04 (1·01–1·07)	139	1·37 (1·16–1·62)	385	1·08 (0·98–1·18)	986	1·06 (0·99–1·13)
All smokers		..	15 223	1·13 (1·11–1·15)	655	1·76 (1·62–1·91)	1425	1·13 (1·07–1·20)	3932	1·25 (1·21–1·30)
Non-smokers		..	4855	1·00 (0·97–1·03)	150	1·00 (0·85–1·18)	499	1·00 (0·91–1·09)	1114	1·00 (0·94–1·06)
Trend p value†				<0·0001		<0·0001		0·0620		<0·0001

Group-specific CI for the non-smoker RR of 1·00 reflects the variance of the log risk in non-smokers; for each of the other RRs the CI is correspondingly wider than the group-specific CI. COPD=chronic obstructive pulmonary disease.

* RRs (smokers *vs* non-smokers) were adjusted for 5-year age group and region, alcohol, and education; additional adjustment for occupation made no material difference to the RRs.

† Trend test in smokers, ignoring non-smokers; if trend tests in this table had included non-smokers, each would have yielded p<0·0001.

**Table 2:** Amount last smoked versus cause-specific mortality rate ratio (RR) among urban and rural men in about 2010 (second study) and about 1995 (first study)

		**Breath CO, ppm**	**All causes**		**Lung cancer**		**Ischaemic stroke or ischaemic heart disease**		**COPD**	
			Number of deaths	RR* (95% group-specific CI)	Number of deaths	RR* (95% group-specific CI)	Number of deaths	RR* (95% group-specific CI)	Number of deaths	RR* (95% group-specific CI)
**Urban men, about 2010 (2006–14)**										
Daily amount smoked (mean)										
	<15 (8·3)	12·0	890	1·48 (1·39–1·59)	115	2·28 (1·90–2·74)	209	1·36 (1·19–1·56)	26	2·94 (1·99–4·35)
	15–24 (19·2)	16·2	1091	1·70 (1·60–1·80)	167	3·28 (2·82–3·83)	261	1·73 (1·53–1·96)	45	5·40 (4·04–7·23)
	≥25 (34·9)	17·6	413	1·93 (1·75–2·12)	68	4·12 (3·24–5·24)	108	2·24 (1·85–2·71)	20	7·26 (4·66–11·32)
All smokers		14·9	2394	1·65 (1·59–1·73)	350	2·98 (2·66–3·33)	578	1·63 (1·49–1·77)	91	4·61 (3·71–5·71)
Non-smokers		4·5	924	1·00 (0·94–1·07)	69	1·00 (0·79–1·27)	242	1·00 (0·88–1·14)	18	1·00 (0·62–1·60)
Trend p value†				<0·0001		<0·0001		<0·0001		0·0021
**Rural men, about 2010 (2006–14)**										
Daily amount smoked (mean)										
	<15 (7·7)	12·1	3298	1·25 (1·20–1·29)	202	1·81 (1·57–2·09)	537	1·27 (1·16–1·39)	347	1·52 (1·36–1·70)
	15–24 (19·3)	14·5	3113	1·17 (1·13–1·22)	333	2·38 (2·14–2·65)	380	1·14 (1·03–1·27)	306	1·32 (1·18–1·47)
	≥25 (35·3)	14·0	1521	1·27 (1·20–1·34)	188	3·20 (2·75–3·72)	161	1·37 (1·16–1·61)	156	1·34 (1·13–1·59)
All smokers		13·6	7932	1·22 (1·20–1·25)	723	2·30 (2·13–2·48)	1078	1·24 (1·17–1·32)	809	1·41 (1·31–1·51)
Non-smokers		6·0	2031	1·00 (0·96–1·05)	92	1·00 (0·81–1·23)	316	1·00 (0·89–1·12)	172	1·00 (0·86–1·16)
Trend p value†				0·7875		<0·0001		0·9951		0·1126
**Urban men, about 1995 (1991–99)**										
Daily amount smoked (mean)										
	<15 (8·2)	..	1332	1·23 (1·16–1·30)	104	1·77 (1·46–2·15)	202	1·22 (1·06–1·40)	223	1·28 (1·12–1·46)
	15–24 (19·2)	..	1660	1·35 (1·29–1·42)	190	2·62 (2·27–3·03)	261	1·40 (1·24–1·59)	256	1·45 (1·28–1·64)
	≥25 (36·1)	..	660	1·51 (1·39–1·63)	75	2·80 (2·21–3·53)	101	1·55 (1·27–1·89)	110	1·78 (1·46–2·15)
All smokers		..	3652	1·32 (1·28–1·37)	369	2·32 (2·08–2·59)	564	1·35 (1·23–1·47)	589	1·42 (1·30–1·55)
Non-smokers		..	1381	1·00 (0·95–1·06)	82	1·00 (0·80–1·25)	240	1·00 (0·87–1·14)	197	1·00 (0·87–1·15)
Trend p value†				<0·0001		0·0015		0·0389		0·0071
**Rural men, about 1995 (1991–99)**										
Daily amount smoked (mean)										
	<15 (8·9)	..	3959	1·16 (1·09–1·16)	132	1·37 (1·15–1·63)	434	1·10 (1·00–1·21)	1005	1·25 (1·17–1·33)
	15–24 (18·8)	..	6886	1·13 (1·10–1·15)	327	1·90 (1·70–2·12)	659	1·18 (1·09–1·28)	1800	1·23 (1·18–1·29)
	≥25 (36·5)	..	4378	1·13 (1·10–1·17)	196	1·84 (1·58–2·13)	332	1·08 (0·97–1·21)	1127	1·29 (1·22–1·38)
All smokers		..	15 223	1·13 (1·11–1·15)	655	1·76 (1·62–1·91)	1425	1·13 (1·07–1·20)	3932	1·25 (1·21–1·30)
Non-smokers		..	4855	1·00 (0·97–1·03)	150	1·00 (0·85–1·18)	499	1·00 (0·91–1·09)	1114	1·00 (0·94–1·06)
Trend p value†				0·7163		0·0233		0·8946		0·4014

Daily amount smoked adds together cigarettes plus g of other tobacco. Group-specific CI for the non-smoker RR of 1·00 reflects the variance of the log risk in non-smokers. COPD=chronic obstructive pulmonary disease.

* RRs (smokers *vs* non-smokers) were adjusted for 5-year age group and region, alcohol, and education; additional adjustment for occupation made no material difference to the RRs.

† Trend test in smokers, ignoring non-smokers; if trend tests in this table had included non-smokers, each would have yielded p<0·0001.

**Table 3:** Deaths attributed to tobacco in China, 2010

	**Male**	**Female**	**Both**
30–69 years	375/2030	20/968	395/2998
70–79 years	250/1370	45/907	295/2277
≥80 years*	215/1178	65/1269	280/2447
All ages	840/4578	130/3144	970/7722

Data are number of deaths caused by tobacco (thousands)/total number of deaths (thousands). Below age 30 years, the total number of deaths (thousands) was 398 in men and 200 in women.

* Estimated indirectly by applying mortality rate ratios at age 70–79 years.
