# Supplementary material for: Contrasting male and female trends in tobacco-attributed mortality in China: evidence from successive nationwide prospective cohort studies
Source: Lancet. 2015 Oct 10;386(10002):1447–56. doi: 10.1016/S0140-6736(15)00340-2 (PMC4691901; doi:10.1016/S0140-6736(15)00340-2)
Supplement: Supplementary appendix [file mmc1.pdf]

THE LANCET

Supplementary appendix

This appendix formed part of the original submission and has been peer reviewed. We post it as supplied by the authors.

Supplement to: Chen Z, Peto R, Zhou M, et al, for the China Kadoorie Biobank (CKB) collaborative group. Contrasting male and female trends in tobacco-attributed mortality in China: evidence from successive nationwide prospective cohort studies. *Lancet* 2015; **386:** 1447–56.

Online Appendix contents list; click on any item to jump to it

Page 3
Webtable 1: ICD codes for specific causes of death

Page 4
Webtable 2: Numbers of deaths at age 40-79, RRs and proportions of deaths attributed to smoking among men in the first (CPSS, 1991-99) and men and women in the second study (CKB, 2006-14)

Page 5
Webtable 3: Baseline characteristics of men at age 40-79 in the first (CPSS, 1991) versus second study (CKB, 2004-08)

Page 6
Webfigure 1: Adjusted rate ratios for all-cause mortality in men, by area, in the second study (CKB, 2006-14)

Page 7
Webfigure 2: Ex- versus never-smoker all-cause mortality rate ratio, by years stopped smoking and reason stopped, for men in both studies combined

Page 8
Webfigure 3: Changing Chinese male lung cancer mortality, 1995-2010, among ever-smokers and never-smokers in two nationally representative prospective studies about 15 years apart

Page 9
Webfigure 4: Chinese never-smoker lung cancer death rates by age and sex in two successive prospective studies 15 years apart (1991-99 and 2006-14)

Page 10
Webfigure 5: US never-smoker lung cancer mortality rates by age and sex in the American Cancer Society's Second Cancer Prevention Study, CPS-II, during years 3-6 of the prospective follow-up (approximately 1984-88)

Webtable 1: ICD codes for specific causes of death _

Category	ICD-9	ICD-10
Lung cancer (including trachea)	162	C33-C34
Liver cancer	155	C22
Stomach cancer	151	C16
Oesophagus cancer	150	C15
Five minor sites (Lip/oral cavity, larynx, pharynx, pancreas and bladder)	140-149, 157, 161, 188	C00-C14, C25, C32, C67
Cancer, other or unknown	Rest of 140-208	Rest of C00-C97
**All cancer**	**140-208**	**C00-C97**
Ischaemic heart disease	410-414	I20-I25
Ischaemic stroke	433-435	I63
Haemorrhagic stroke	430-432	I61
Other or unknown type of stroke	436-438	Rest of I60-I69
Other cardiovascular	Rest of 410-438, exc. 415-417	Rest of I00-I99, exc. I26-I27
**All vascular**	**410-438, exc. 415-417**	**I00-I99 exc. I26-I27**
COPD	490-496, 415-417	J40-J44, I26-I27
Other respiratory	Rest of 460-519	Rest of J00-J99
**All respiratory**	**460-519, 415-417**	**J00-J99, I26-I27**
**All other medical**	**Rest of 001-799**	**Rest of A00-R99**
**All non-medical**	**800-999, E800-E999, V01-V82**	**V01-Y98**

Webtable 2: Number of deaths at age 40-79, mortality RRs and proportion of deaths attributed to smoking among men in ~1995 (CPSS, 1991-99) and men and women in ~2010 (CKB, 2006-14)

Cause of deaths	CPSS men only (First study, 1991-99)			CKB men (Second study, 2006-14)			CKB women (Second study, 2006-14)		
	**No of deaths Smokers/Non-smokers***	**RR† (95% CI)**	**PAF‡ (%)**	**No of deaths Smokers/Non-smokers***	**RR† (95% CI)**	**PAF‡ (%)**	**No of deaths Smokers/Non-smokers***	**RR† (95% CI)**	**PAF‡ (%)**
Lung cancer	1024/232	1.95 (1.68 - 2.26)	39	1073/161	2.58 (2.17 - 3.05)	51	101/618	2.56 (2.02 - 3.26)	9
Liver cancer	977/374	1.06 (0.93 - 1.20)	3	644/176	1.26 (1.06 - 1.50)	16	31/382	1.40 (0.93 - 2.12)	2
Stomach cancer	870/273	1.22 (1.06 - 1.41)	14	602/170	1.25 (1.05 - 1.49)	14	26/334	1.43 (0.92 - 2.23)	2
Oesophagus cancer	662/233	1.18 (1.01 - 1.38)	11	567/124	1.58 (1.30 - 1.93)	28	13/236	1.05 (0.55 - 2.01)	0
Five minor sites	215/78	1.18 (0.90 - 1.54)	11	282/62	1.53 (1.15 - 2.04)	27	14/210	0.98 (0.55 - 1.75)	-1
**All cancer**	**4201/1352**	**1.28 (1.20 - 1.36)**	**16**	**3811/913**	**1.51 (1.40 - 1.63)**	**26**	**272/3037**	**1.58 (1.37 - 1.81)**	**3**
IHD	1172/406	1.26 (1.12 - 1.42)	16	1261/426	1.38 (1.23 - 1.54)	19	130/1212	1.74 (1.42 - 2.12)	5
Ischaemic stroke	817/333	1.11 (0.97 - 1.27)	7	395/132	1.41 (1.15 - 1.73)	19	25/353	1.15 (0.74 - 1.78)	1
Haemorrhagic stroke	2437/816	1.06 (0.98 - 1.16)	5	1251/397	1.03 (0.92 - 1.16)	1	88/1262	1.09 (0.86 - 1.39)	1
**All cardiovascular**	**6277/2203**	**1.12 (1.07 - 1.18)**	**8**	**3578/1193**	**1.24 (1.16 - 1.33)**	**13**	**319/3530**	**1.44 (1.27 - 1.63)**	**3**
**All respiratory**	**5193/1504**	**1.27 (1.20 - 1.35)**	**16**	**1068/244**	**1.64 (1.42 - 1.90)**	**31**	**159/710**	**1.78 (1.46 - 2.17)**	**8**
**All other medical**	**2055/752**	**1.07 (0.98 - 1.17)**	**5**	**1062/360**	**1.20 (1.06 - 1.36)**	**12**	**110/1133**	**1.41 (1.13 - 1.76)**	**3**
**All causes**	**18875/6236**	**1.17 (1.14 - 1.21)**	**11**	**10326/2955**	**1.33 (1.28 - 1.39)**	**18**	**897/9037**	**1.51 (1.40 - 1.63)**	**3**

* Excludes 4306 and 15,271 (14,080 male) ever regular smokers who stopped smoking by choice in CPSS and CKB respectively.

† Rate ratios (RRs) were all adjusted for area and age at risk (strata), education and alcohol. Additional adjustment for SBP ((1.18 [1.16-1.20], 1.35 [1.32-1.37] , 1.56 [1.46-1.67] for, respectively CPSS men, CKB men and CKB women), for BMI ((1.15 [1.13-1.17], 1.30 [1.27-1.32], 1.48 [1.39-1.58] for, respectively CPSS men, CKB men and CKB women) and for SBP and BMI ((1.15 [1.13-1.17], 1.30 [1.27-1.33], 1.53 [1.43-1.63] for, respectively CPSS men, CKB men and CKB women) hardly changed the RRs for overall mortality.

‡ In estimating Population Attributable Fraction (PAF) for ever regular smoking, the RR used is for all ever regular smokers including those who stopped by choice.

Webtable 3: Baseline characteristics of men at age 40-79 in the first (CPSS, 1991) versus second study (CKB, 2004-08)

Characteristics	CPSS (n=219,893)	CKB* (n=180,658)
**% urban**	27.1	43.7
**Year of baseline survey (median)**	1991	2006
**Year of birth (median)**	1938	1952
**Age at baseline (%)**		
40-49	37.8	32.8
50-59	31.6	35.3
60-69	21.4	22.9
70-79	9.2	9.1
Mean age (years)	*54.8*	*55.4*
**Highest education (%)**		
None	22.7	10.0
Primary	44.2	35.5
Secondary	27.8	47.4
Tertiary	5.3	7.0
**Current alcohol drinking in most weeks (%)**	33.4	33.2
**Smoking category (%)**		
Never smoked regularly	26.8	24.7
Ex-smoker (stopped by choice)	2.0	7.2
[Ex-smoker (stopped by choice) as % of ever-smoker]	*[2.7]*	*[9.6]*
Smoker (current smoker or stopped because ill)	71.2	68.1
– *Current smoker*	*67.4*	*60.8*
– *Stopped because ill*	*3.8*	*7.3*
**Patterns among smokers #**		
**Age started (%)**		
<20	30.2	32.2
20-24	41.6	37.2
≥25	28.2	30.6
**First started on cigarettes, by birth year (%)**		
<1925	41.5	—
1925-1934	52.0	56.8
1935-1944	60.1	64.0
1945-1954	69.1	75.2
≥1955	—	89.2
**Last smoked cigarettes only, by age (%)**		
40-49	67.8	92.9
50-59	54.9	83.0
60-69	43.2	73.2
70-79	31.5	71.4
Any age	55.4	83.4
Mean cigarettes/day/pure cigarette smoker of any age	*16.9*	*18.1*

* Excluded 29,564 men aged <40 at entry

Current smoker or stopped because ill

Webfigure 1: Adjusted rate ratios for all−cause mortality in men, by area, in the second prospective study (CKB, 2006−14)

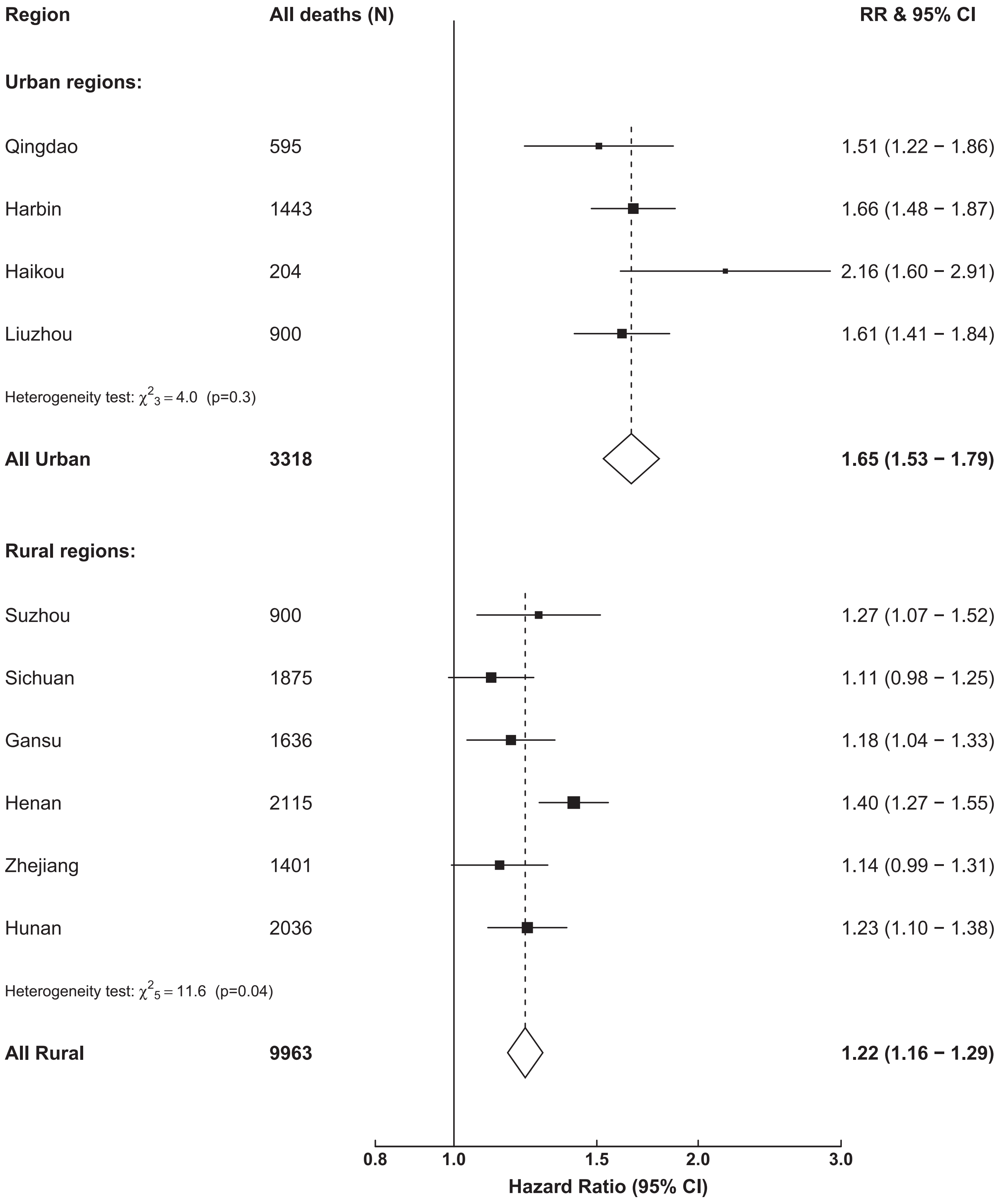

Webfigure 2: **Ex- versus never-smoker all-cause mortality rate ratio, by years stopped smoking and reason stopped; men in both studies**

Each group-specific CI (including that for never-smokers, given by the width of the shaded strip) reflects the variance of the log risk in that 1 group, so comparisons use variances from >1 group.

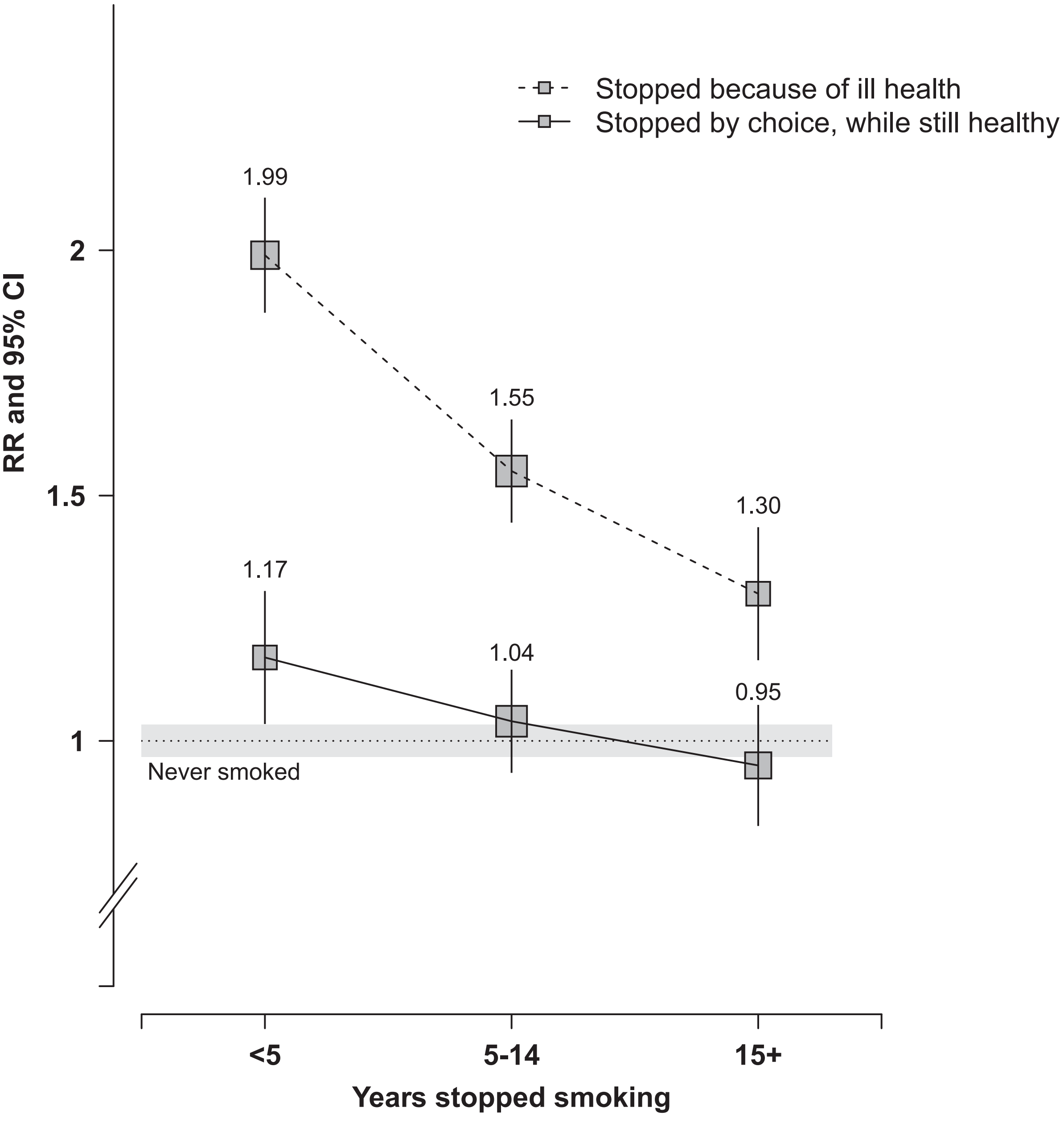

Webfigure 3: **Changing Chinese male lung cancer mortality, 1995-2010, among ever-smokers and never-smokers in two nationally representative prospective studies about 15 years apart**

NB: Rates in later middle age are increasing in Chinese smokers, decreasing in Chinese never-smokers, and roughly constant in US never-smokers. CKB, Chinese Kadoorie Biobank, follow-up ~2006-2014; CPSS, Chinese Prospective Smoking Study, follow-up ~1991-1999; USA, ACS CPS-II, follow-up 1984-1988 [3]. Non-smoker rates: webtables 4-5.

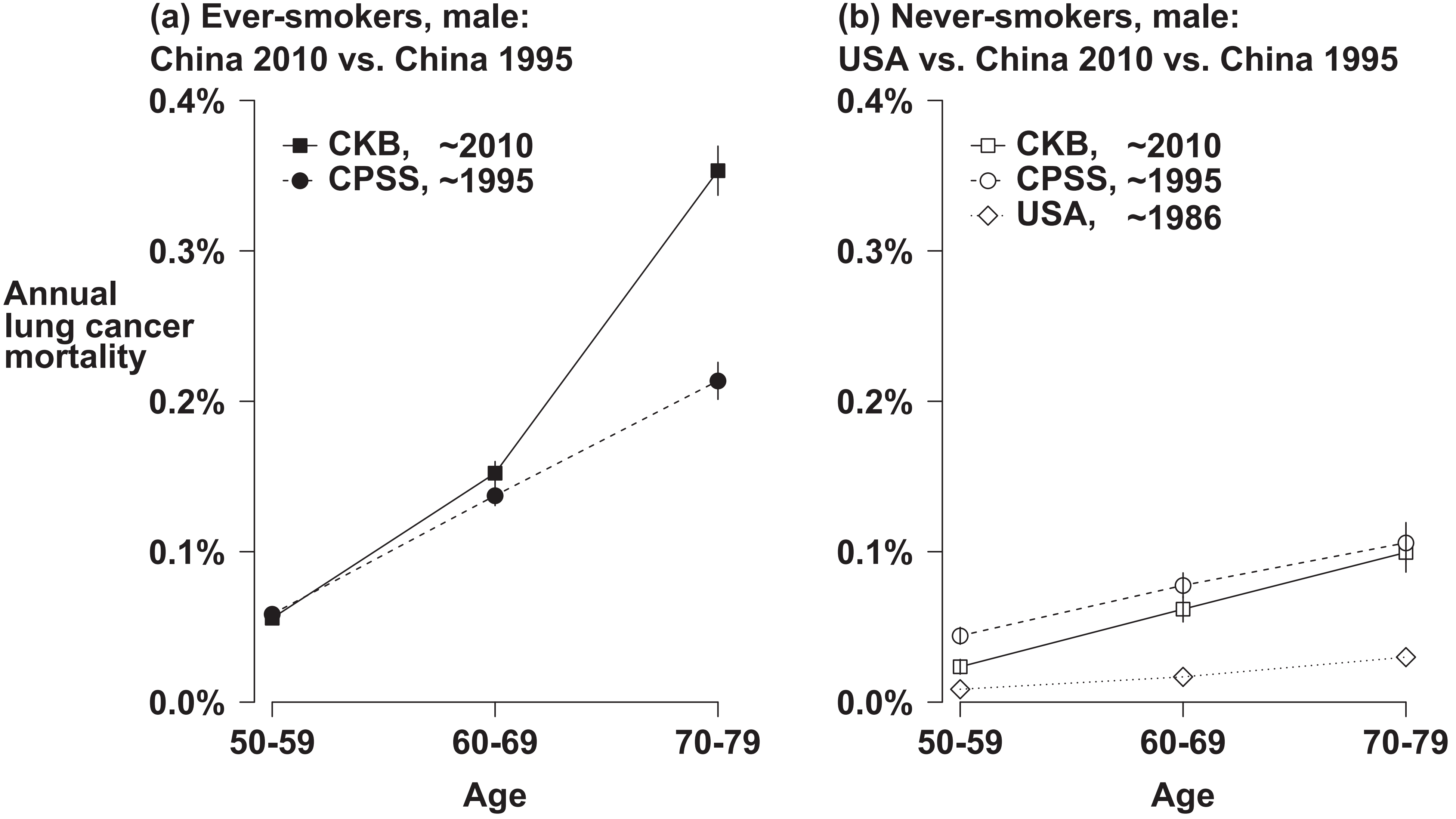

Webtable 4: **Chinese never-smoker lung cancer death rates by age and sex in two successive prospective studies 15 years apart (1991-99 and 2006-14)**

	Male, 1991-99 (mid-year 1995)			Male, 2006-14 (mid-year 2010)			Female, 2006-14 (mid-year 2010)		
**Age at risk (in years)**	**Person years**	**Lung ca deaths**	**Rate / 100000**	**Person years**	**Lung ca deaths**	**Rate / 100000**	**Person years**	**Lung ca deaths**	**Rate / 100000**
**40-44**	32391	5	**18**	56656	6	**10**	321656	10	**8**
**45-49**	76630	16		52234	5		341274	45	
**50-54**	71812	31	**44**	46190	7	**26**	328126	51	**21**
**55-59**	68713	31		51852	19		350710	91	
**60-64**	62924	46	**78**	46243	22	**66**	259817	99	**54**
**65-69**	49204	41		39430	33		176555	123	
**70-74**	35752	34	**109**	36527	37	**125**	131322	116	**107**
**75-79**	22810	28		21490	32		65702	83	
**Total, by summation**	420235	232		350621	161		1975162	618	

Never-smokers include those who reported at baseline having smoked occasionally, but never regularly.

The crude rate in each age group is 100,000 × the number of lung cancer deaths / person-years.

The rate in each 10-year age group is the mean of the two crude rates in the component 5-year age groups.

Webtable 5: US never-smoker lung cancer mortality rates by age and sex in the American Cancer Society's Second Cancer Prevention Study, CPS-II, during years 3-6 of the prospective follow-up (approximately 1984-88)

Age at risk (in years)	Male, 1984-88 (mid-year 1986)			Female, 1984-88 (mid-year 1986)		
	Person years	Annual lung cancer mortality / 100,000		Person years	Annual lung cancer mortality / 100,000	
		Crude rate	Smoothed		Crude rate	Smoothed
**35-39**	14560	7	**2**	30810	3	**2**
**40-44**	13930	0	**3**	45760	0	**3**
**45-49**	32110	9	**5**	116870	4	**4**
**50-54**	81410	5	**7**	196520	5	**7**
**55-59**	87920	3	**10**	224110	8	**10**
**60-64**	80570	11	**14**	227400	14	**14**
**65-69**	69670	24	**20**	192560	18	**19**
**70-74**	50530	36	**27**	143280	28	**26**
**75-79**	28880	38	**35**	95900	42	**34**

Source: Appendix to Reference (3). Never-smoker means replied "never smoked regularly" at baseline survey.

The crude rate in each age group is 100,000 × the number of lung cancer deaths / person-years.

Smoothing the lung cancer mortality rates in the 5-year age groups with lower limits 35, 40, … , 75 involved obtaining the best-fitting line of slope 4 on a logarithmic graph of the crude rates vs these lower age limits; then, in Webfigure 3, the US rates at ages 50-59, 60-69 and 70-79 are each the mean of two smoothed rates.
